# The relationship between problem-solving appraisal and internalizing disorders: a meta-analysis

**DOI:** 10.3389/fpsyg.2026.1824501

**Published:** 2026-05-18

**Authors:** Tyrone B. Pretorius, Anita Padmanabhanunni

**Affiliations:** 1 Department of Psychology, University of the Western Cape, Cape Town, South Africa; 2 Monash University, Melbourne, VIC, Australia

**Keywords:** anxiety, depression, internalizing disorders, meta-analysis, problem-solving appraisal

## Abstract

**Background:**

Problem-solving appraisal is an important cognitive process that influences how individuals manage and cope with stress. Negative appraisals of problem-solving ability are associated with adverse mental health outcomes.

**Methods:**

We conducted a meta-analysis using a random-effects model to synthesize the relationship between problem-solving appraisal and internalizing disorders. In addition, we examined the potential moderating role of age in this relationship and conducted subgroup analyses. To account for effect size dependencies, we used robust variance estimation (RVE). We examined publication bias with a funnel plot, Egger’s regression test, and weighting studies in terms of their probability of being published.

**Results:**

We found a medium combined effect size (*r* = 0.40) for 66 coefficients from 49 studies. Age did not moderate the combined effect size. Subgroup analyses found no difference between the combined effect size of studies that used a translated version of the PSI and those that used the original English version, nor was there a difference between those who reported on the relationship between problem-solving appraisal and anxiety and those that focused on the relationship between problem-solving appraisal and depression. The combined effect size obtained with RVE did not significantly differ from the original combined effect size. A funnel plot, Egger’s regression test, and weighting of studies in terms of their probability of being published all evidenced publication bias. The trim-and-fill method imputed 16 studies, which adjusted the obtained combined effect size to a medium effect size of 0.38.

**Conclusion:**

The findings underscore the moderate but robust relationship between problem-solving appraisal and internalizing disorders. Interventions to improve problem-solving skills may be beneficial across various demographics. Addressing publication bias is crucial to ensuring accurate meta-analytic results in mental health research.

## 1 Introduction

Mood and anxiety disorders are among the most prevalent mental health disorders globally. Depression is characterized by feelings of sadness and loss of interest and pleasure in daily activities, and especially cognitive (e.g., thoughts of suicide) and somatic (e.g., weight loss) alterations that adversely impact the individual’s social, occupational, and interpersonal functioning (American Psychiatric Association, 2022). Anxiety is associated with physical and cognitive symptoms such as irritability, difficulty controlling worry, disturbances of sleep and concentration, restlessness, and irritability (American Psychiatric Association, 2022). Research within longitudinal studies and nonclinical samples of adults and adolescents with depressive and anxiety disorders support a developmental progression from anxiety to depression (Danneel et al., 2020; Bendau et al., 2021; del-Valle et al., 2022; Kenntemich et al., 2024). Depression is associated with a mental focus on past experiences, particularly those involving the loss of important goals or aspirations. Individuals experiencing depression tend to dwell on these lost goals, which can result in a pattern of negative and biased cognitive attributions of the self and future. In contrast, anxiety is associated with a future orientation and is linked to the perception of a future threat (Beck et al., 1974; Kenntemich et al., 2024).

Although anxiety and depression frequently co-occur, the reasons for this comorbidity are unclear. Some researchers have suggested that anxiety may precipitate cognitive processes that lead to depressive symptoms, including ruminative thinking, brooding, and cognitions associated with hopelessness. Rumination entails repeatedly focusing on negative emotional experiences and their causes or consequences, whereas brooding involves a passive comparison of one’s current life situation or status with an unachieved and idealized standard (Starr and Davila, 2012; Eysenck and Fajkowska, 2018). The tripartite model (Clark and Watson, 1991) has been influential in accounting for the co-occurrence of anxiety and depression. This model proposes that anxiety and depression share a common valence (negative emotions and the experience of distress) that underlies their comorbidity. The model also proposes that there are three core components of these disorders: general distress (negative affect), which is specific to both anxiety and depression; physiological hyperarousal, which is more characteristic of anxiety than depression; and anhedonia, which is more typical of depression than anxiety. The presence of general distress or negative affect means that individuals who experience one of these conditions are also likely to suffer from the other (Clark and Watson, 1991).

A consistent finding that has emerged from research on mental health disorders is that the burden of mental illness is not equally distributed in the population. Rather, it is concentrated in a subset of individuals. This finding has prompted research into the factors underlying this differential vulnerability (Zimmermann et al., 2020; Padmanabhanunni and Wiid, 2022; Shoshani and Kor, 2022). According to Lazarus and Folkman’s transactional model of stress and coping, cognitive appraisals are central in influencing mental health outcomes. Their model differentiates between primary appraisals, which entail the initial determination of whether a stressor is perceived as benign or stressful, and secondary appraisals, which involve an assessment of the resources the individual has available for coping (Lazarus and Folkman, 1984).

The current study focuses on the relationship between problem-solving appraisal and internalizing disorders. Problem-solving appraisal refers to an individual’s subjective assessment of their ability to solve life problems effectively (Heppner, 1982; Heppner, 1988). This concept is similar to Lazarus and Folkman’s construct of secondary appraisal. Self-appraisals of competence and coping ability influence individuals’ emotional reactions to stressors and subsequent development of psychological distress. In a review of 20 years of research on the Problem-Solving Inventory (PSI), which assesses problem-solving appraisal, Heppner et al. (2004) concluded that problem-solving appraisal is a critical determinant of psychological adjustment and mental health outcomes. Their findings indicate that individuals who perceive themselves as more effective problem solvers tend to experience lower levels of stress and mental health disorders. Conversely, those with negative appraisals of their own problem-solving abilities are more likely to exhibit higher levels of internalizing disorders.

Suzuki and Ahluwalia (Suzuki and Ahluwalia, 2004) have noted several methodological concerns with Heppner et al. (2004)’ work. First, the quality of the reported data varied, particularly in terms of effect sizes (e.g., correlations) and sample descriptors (e.g., race/ethnicity, sample size, and gender). Suzuki and Ahluwalia argue that these inconsistencies highlight the need for a meta-analysis of the PSI to provide a more accurate and comprehensive understanding of the relationship between problem-solving appraisal and mental health outcomes. Such an analysis would address concerns regarding uneven reporting and offer robust conclusions about the impact of problem-solving appraisal across diverse populations. Hence, the current study is a meta-analysis that aims to synthesize the relationship between problem-solving appraisal, as measured by the PSI, and internalizing disorders, specifically depression and anxiety.

## 2 Method

### 2.1 Procedure

The meta-analysis followed the guidelines of the Preferred Reporting Items for Systematic Reviews and Meta-Analyses (PRISMA: Moher et al., 2009). We aimed to find studies that examine the connection between problem-solving appraisal, assessed by the PSI, and internalizing disorders, specifically depression and anxiety. To this end, we searched PubMed, PsycArticles, ScienceDirect, Scopus, Taylor & Francis, and Web of Science for studies published from 1982, when the PSI was first published, to November 2023. The same search strategy was applied across databases, using the keywords “problem-solving appraisal” and “problem-solving inventory,” with minor database-specific formatting adjustments where necessary. The search was limited to English language records.

The two authors independently reviewed the abstracts of the identified studies. For inclusion, studies needed to be in English, utilize the PSI to evaluate problem-solving appraisal, and report on the relationship of problem-solving appraisal and internalizing disorders. We obtained full-text versions for all abstracts that were tentatively considered to meet the inclusion criteria. In cases of disagreement over an abstract, the full text was reviewed. Additionally, we used a snowballing technique to identify further studies from the reference lists of reviewed papers.

### 2.2 Data analysis

A random-effects model was used for the meta-analysis. The meta-analysis was conducted using the “metafor” (Viechtbauer, 2010) package in R (R Development Core Team, 2020). Heterogeneity of effect sizes was evaluated using I^2^, where 25%: = low heterogeneity, 50% = moderate heterogeneity, 75% = substantial heterogeneity (Higgins and Thompson, 2002). To evaluate publication bias, we utilized funnel plots and Egger’s regression test. A symmetrical distribution of effect sizes around the combined effect size in the funnel plot and a nonsignificant result from Egger’s test would suggest no publication bias. If effect sizes were asymmetrically distributed around the combined effect size (thus indicating publication bias), we used the trim-and-fill method to impute effect sizes and make the distribution symmetrical. In this instance, the trim-and-fill method provides an adjusted combined effect size to account for the missing effect sizes. We used Meta-Essentials (Suurmond et al., 2017) for the trim-and-fill method. However, funnel plots and Egger’s regression test are used primarily to detect small study bias, which can encompass publication bias (Quintana, 2015); therefore, we also assessed publication bias by applying weights to studies based on their publication probability, using the weight function in R. The combined effect size obtained when studies are weighted is then compared to the unweighted combined effect size using chi-square. A statistically significant chi-square would be indicative of publication bias.

To determine whether other study variables impacted the combined effect size, we examined the potential moderating role of age using meta-regression. We also conducted subgroup analyses of studies that focused on anxiety versus studies that focused on depression and studies that used the original English version of the PSI in comparison with studies that used a translated version of the PSI.

Meta-analysis assumes that effect sizes are independent. However, multiple studies in this analysis reported several effect sizes. To address the dependencies among these effect sizes, we employed robust variance estimation (RVE) with a small sample correction (Hedges et al., 2010). By comparing the combined effect size from RVE with the initial combined effect size, we determined the impact of effect size dependencies on the overall results. The RVE was conducted with the package “robumeta” (Fisher and Tipton, 2015) in the R software environment (R Development Core Team, 2020).

## 3 Results

### 3.1 Studies selection and characteristics

**Figure 1:**
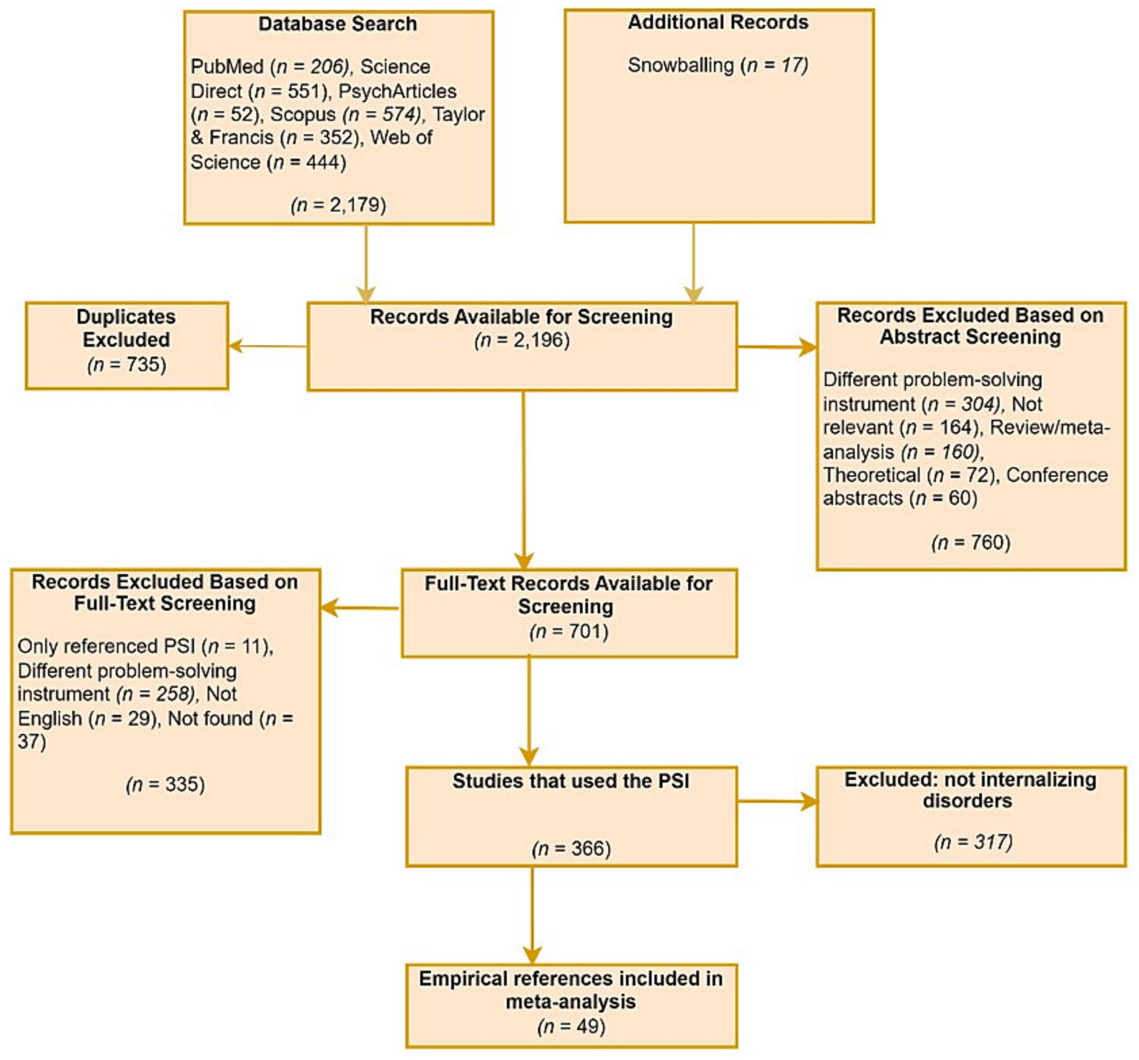
PRISMA diagram of study selection for the meta-analysis of the relationship between problem-solving appraisal and internalizing disorders.

Figure 1 presents the PRISMA flow diagram for the study selection process. A total of 2,196 studies were initially identified. Of these, 366 used the PSI, while the remainder were excluded for reasons such as duplication and not being published in English. Among the 366 studies that used the PSI, 317 did not examine the relationship between problem-solving appraisal and internalizing disorders. The final meta-analysis therefore included 49 studies that specifically investigated this relationship. The characteristics of the included studies are presented in Table 1.

**Table 1:** Characteristics of studies extracted for use in the meta-analysis of the relationship between the PSI and internalizing disorders.

Author(s) & year	Population	*n*	Age	Language	Outcome	*r*
Pretorius et al. (2023)	Students	322	26.0	English	Depression	0.47
Teo et al. (2021)	Young males	342	20.4	English	Depression	0.40
Pretorius (2020)	Secondary school teachers	207	33.0	English	Anxiety	0.52
Korkmaz et al. (2020a)	Individuals who had attempted suicide	50	35.3	Translated	Depression	0.68
Korkmaz et al. (2020a)	Individuals who had attempted suicide	50	35.3	Translated	Anxiety	0.65
Korkmaz et al. (2020b)	Healthcare workers	140	33.2	Translated	Anxiety	0.18
Ji et al. (2018)	Caregivers of individuals with memory loss	78	66.5	English	Depression	0.32
Julal (2016)	Students	175	20.5	English	Depression	0.22
Tang et al. (2015)	Caregivers of adults with memory loss	91	67.0	English	Depression	0.23
Cha et al. (2015)	Young adults	96	24.1	English	Depression	0.38
Chow and Chan (2010)	Chinese migrant women	68		Translated	Depression	0.43
Erdur-Baker (2009)	High school students	250	15.4	Translated	Depression	0.22
Eskin et al. (2008)	High school students	805	14.8	Translated	Depression	0.30
Pickett Jr et al. (2007)	Caregivers of persons with Huntington’s disease	62	55.4	English	Depression	0.27
Pickett Jr et al. (2007)	Persons with Huntington’s disease	61	51.6	English	Depression	0.39
Yeung et al. (2007)	Caregivers of stroke patients	70	N/A	Translated	Depression	0.35
Heppner et al. (2006)	Students	2,889	20.7	Translated	Depression	0.32
Heppner et al. (2006)	Students	2,889	20.7	Translated	Anxiety	0.22
Eremsoy et al. (2005)	Graduate students	92	26.1	Translated	Depression	0.34
Eremsoy et al. (2005)	Graduate students	92	26.1	Translated	Anxiety	0.46
Bastian et al. (2005)	Students	246	19.9	English	Anxiety	0.29
Good et al. (2004)	Students	260	N/A	English	Depression	0.24
Good et al. (2004)	Students	260	N/A	English	Anxiety	0.31
Dieserud et al. (2003)	Suicide attempters	50	41.0	Translated	Depression	0.50
Dieserud et al. (2002)	Individuals who had attempted suicide	321	39.0	Translated	Depression	0.63
Heppner et al. (2002)	Students	234	23.3	English	Depression	0.23
Heppner et al. (2002)	Students	234	23.3	English	Anxiety	0.31
Kerns et al. (2002)	Patients with chronic pain	234	50.0	English	Depression	0.38
Dieserud et al. (2001)	Patients with and without suicide attempts	123	40.2	Translated	Depression	0.48
Witty et al. (2001)	Persons with chronic low back pain	78	48.4	English	Depression	0.42
Chan (2001)	Students	499	26.2	English	Depression	0.44
Dixon (2000)	Students	66	20.0	English	Depression	0.17
Dixon (2000)	Students	66	20.0	English	Depression	0.39
Reid and Dixon (2000)	Students	118	30.0	English	Depression	0.67
Marcotte et al. (1999)	Adolescents	306	15.3	Translated	Depression	0.53
Hanson and Mintz (1997)	Older adults	97	77.3	English	Anxiety	0.25
Hanson and Mintz (1997)	Older adults	97	77.3	English	Depression	0.20
Perez et al. (1997)	Male delinquent adolescents	146	16.5	English	Depression	0.41
Miner and Dowd (1996)	Students	278	35.2	English	Depression	0.41
Miner and Dowd (1996)	Students	278	35.2	English	Anxiety	0.42
Mayo and Tanaka-Matsumi (1996)	Dysphoric and non-dysphoric students	28	N/A	English	Depression	0.82
Pretorius and Diedricks (1994)	Students	450	24.1	English	Depression	0.32
Dixon et al. (1993)	Students	277	20.1	English	Depression	0.33
Dixon et al. (1993)	Students	277	20.1	English	Depression	0.43
McGrath and Ratliff (1993)	Students	269	21.5	English	Depression	0.35
McGrath and Ratliff (1993)	Students	269	21.5	English	Depression	0.52
McGrath and Ratliff (1993)	Students	269	21.5	English	Anxiety	0.49
McGrath and Ratliff (1993)	Students	269	21.5	English	Anxiety	0.41
Priester and Clum (1993)	Students	303	N/A	English	Depression	0.30
Sahin et al. (1993)	Students	224	19.8	Translated	Depression	0.33
Sahin et al. (1993)	Students	224	19.8	Translated	Anxiety	0.45
Elliott et al. (1992)	Students	104	25.0	English	Depression	0.41
Elliott et al. (1992)	Participants with spinal cord injuries	102	39.0	English	Depression	0.42
Pretorius (1992)	Students	450	24.0	English	Depression	0.31
Bonner and Rich (1992)	Correctional inmates	146	28.0	English	Depression	0.52
Elliott et al. (1991)	Persons with spinal cord injury	90	32.6	English	Depression	0.50
Larson et al. (1990)	Students	206	N/A	English	Depression	0.44
Larson et al. (1990)	Students	206	N/A	English	Anxiety	0.42
Larson et al. (1990)	Students	206	N/A	English	Anxiety	0.51
Larson et al. (1990)	Students	237	N/A	English	Depression	0.25
Larson et al. (1990)	Students	237	N/A	English	Anxiety	0.35
Larson et al. (1990)	Students	237	N/A	English	Anxiety	0.46
Bonner and Rich (1988)	Students	186	N/A	English	Depression	0.58
Nezu (1986)	Students	310	24.3	English	Anxiety	0.39
Nezu (1986)	Students	310	24.3	English	Anxiety	0.51
Nezu et al. (1986)	Students	128	22.7	English	Depression	0.41

N/A, data not reported.

Across the 49 studies, 66 correlation coefficients were available for analysis. The studies included a total of 18,834 participants, with a mean sample size of 285.4 (SD = 481.7; range = 28–2,889). The mean participant age across studies was 30.9 years (SD = 15.3; range = 14.8–77.3). The included effect sizes were geographically diverse, although they were concentrated primarily in the USA. Of the 66 coefficients, 24 (36.4%) were derived from studies conducted in the USA, 6 (9.1%) from Turkey, and 5 (7.6%) from South Africa. Smaller numbers of coefficients were contributed by studies from Hong Kong and Norway (*n* = 3 each, 4.5%), and from Australia, Canada, Singapore, Taiwan, and the UK (*n* = 1 each, 1.5%). Country information was not available for 20 coefficients (30.3%).

### 3.2 Combined effect size

**Figure 2:**
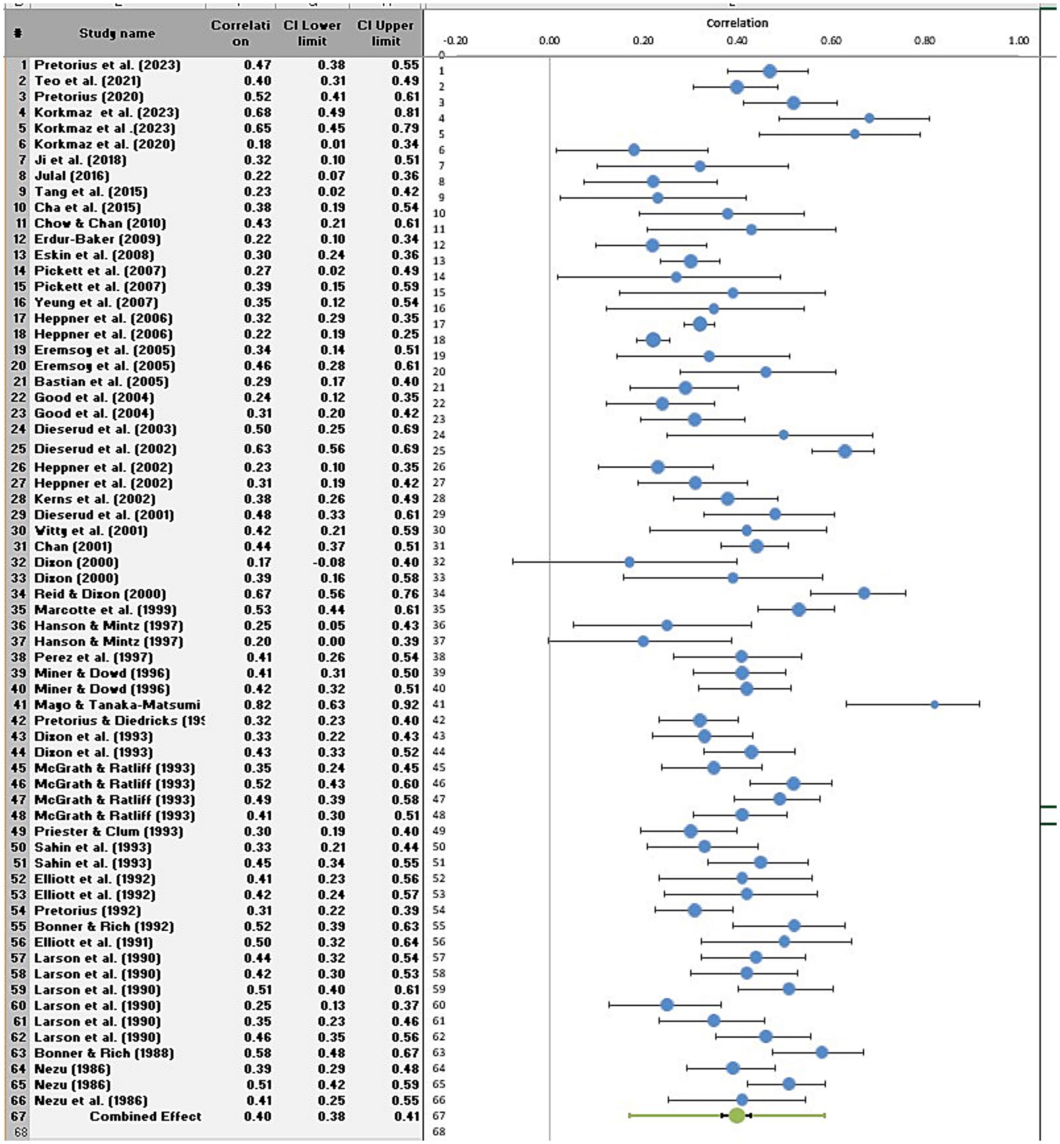
Forest plot of the relationship between problem-solving appraisal and internalizing disorders.

Figure 2 presents the forest plot for the relationship between problem-solving appraisal and internalizing disorders. The pooled effect size across all included studies was 0.40 (95% CI [0.38, 0.41]), indicating a medium association between more negative problem-solving appraisal and higher levels of internalizing disorders. Study weights ranged from 0.59 to 2.11%, suggesting that no single study exerted undue influence on the combined effect size. However, *I*^2^ = 80.04% indicating substantial heterogeneity of effect sizes.

### 3.3 Moderator analysis

Meta-regression was used to examine whether mean age potentially moderated the relationship between problem-solving appraisal and internalizing disorders. The results indicate that age was not a significant moderator of this association (*B* = −0.001, *z* = −1.11, *p* = 0.27).

### 3.4 Subgroup analyses

**Figure 3:**
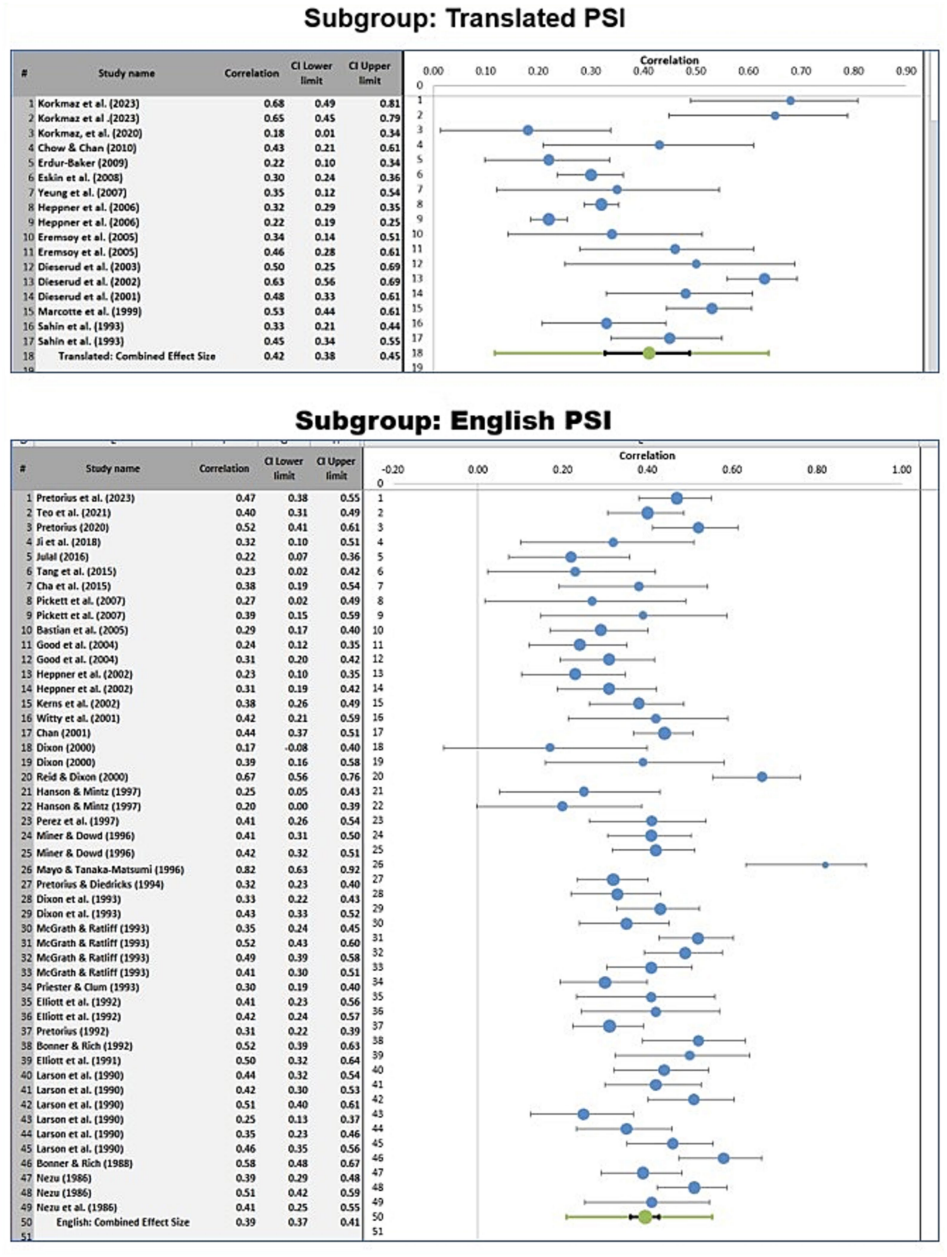
Forest plots of the relationship between problem-solving appraisal and internalizing disorders for studies that used a translated version versus studies that used an English version of the PSI.

**Figure 4:**
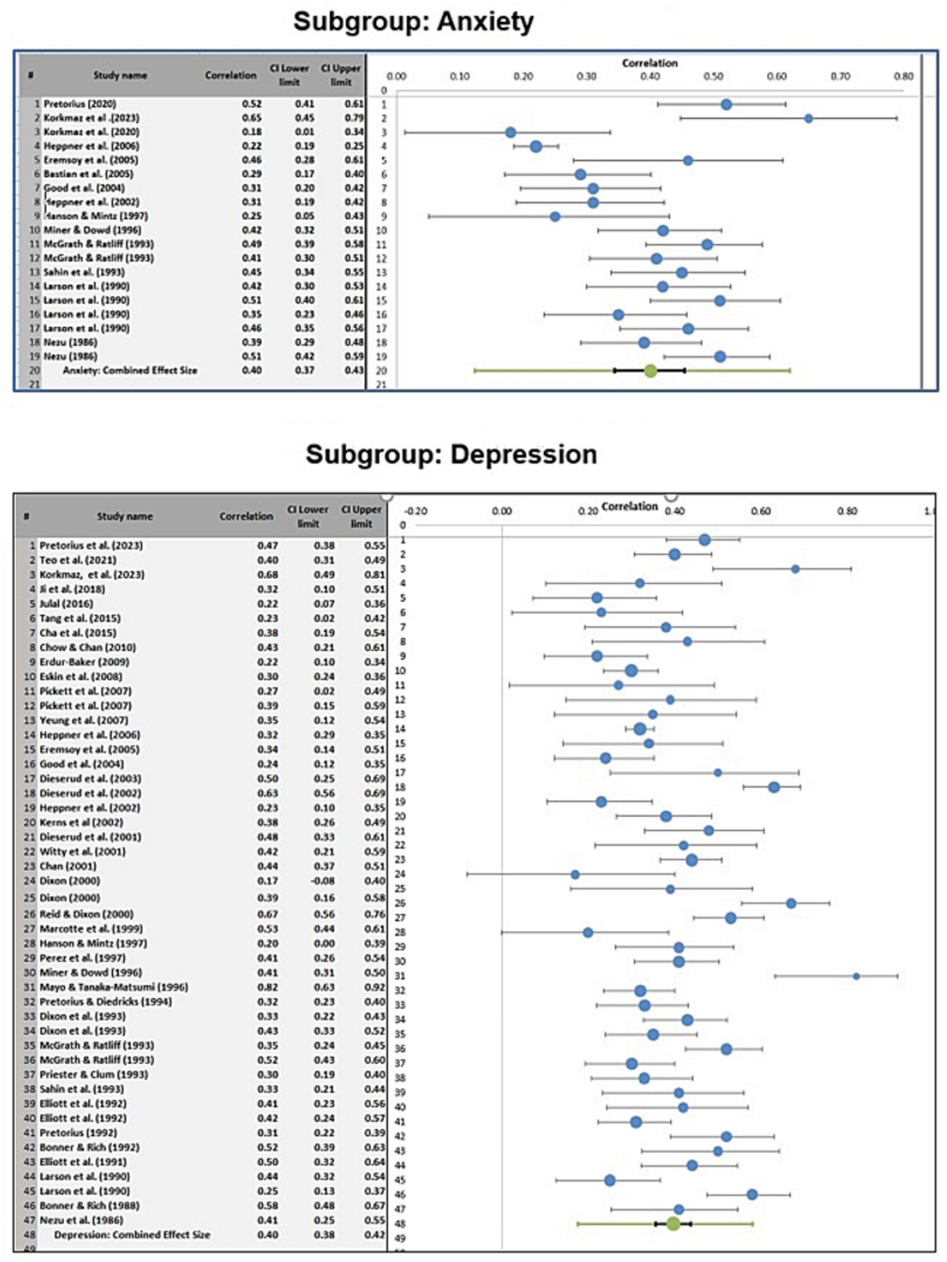
Forest plots of the relationship between problem-solving appraisal and internalizing disorders for studies that focused on anxiety versus studies that focused on depression.

Subgroup analyses were conducted to determine whether the pooled effect size differed according to the language version of the PSI used or according to the specific internalizing disorder outcome examined. Figures 3 and 4 present the relevant forest plots, and Table 2 provides the statistical comparisons of subgroup differences.

**Table 2:** Comparison of combined effect sizes (*r* and 95% confidence intervals for different subgroups.

Subgroup	*n*	Combined effect	95% CI		*z*	*p*
			LL	UL		
English version of PSI	49	0.39	0.37	0.41	−0.60	0.55
Translated version of PSI	17	0.42	0.38	0.45		
PSI and anxiety	19	0.40	0.37	0.43	0.00	1.00
PSI and depression	47	0.40	0.38	0.42		

For studies that used the original English version of the PSI, the pooled effect size was 0.39 (95% CI [0.37, 0.41]). For studies that used a translated version of the PSI, the pooled effect size was 0.42 (95% CI [0.38, 0.45]). This difference was not statistically significant (z = −0.60, *p* = 0.55), indicating that the observed association did not differ meaningfully by language version of the PSI.

A second subgroup analysis compared studies examining anxiety with those examining depression. The pooled effect size for studies focusing on anxiety was 0.40 (95% CI [0.37, 0.43]), while the pooled effect size for studies focusing on depression was also 0.40 (95% CI [0.38, 0.42]). The difference between these subgroup estimates was not statistically significant (z = 0.00, *p* = 1.00), suggesting that the magnitude of the association was similar across these two internalizing outcomes.

### 3.5 Accounting for effect size dependencies

Since multiple effect sizes were available from some studies, RVE was used to account for effect size dependencies. The RVE analysis yielded a pooled effect size of 0.40 (95% CI [0.36, 0.43]), which was substantively identical to the original pooled effect size of 0.40 (95% CI [0.38, 0.41]). The difference between the two estimates was not statistically significant (z = 0.00, *p* = 1.00), indicating that dependence among effect sizes did not materially affect the findings.

### 3.6 Publication Bias

Multiple analyses indicated the presence of publication bias. Egger’s regression test was significant (*z* = 2.33, *p* = 0.02), suggesting asymmetry in the distribution of effect sizes. In addition, weighting studies according to their probability of being published produced a pooled effect size of 0.38 (95% CI [0.35, 0.42]), which differed significantly from the unweighted pooled effect size of 0.40 (95% CI [0.38, 0.41]; χ^2^ = 4.57, *p* = 0.032).

**Figure 5:**
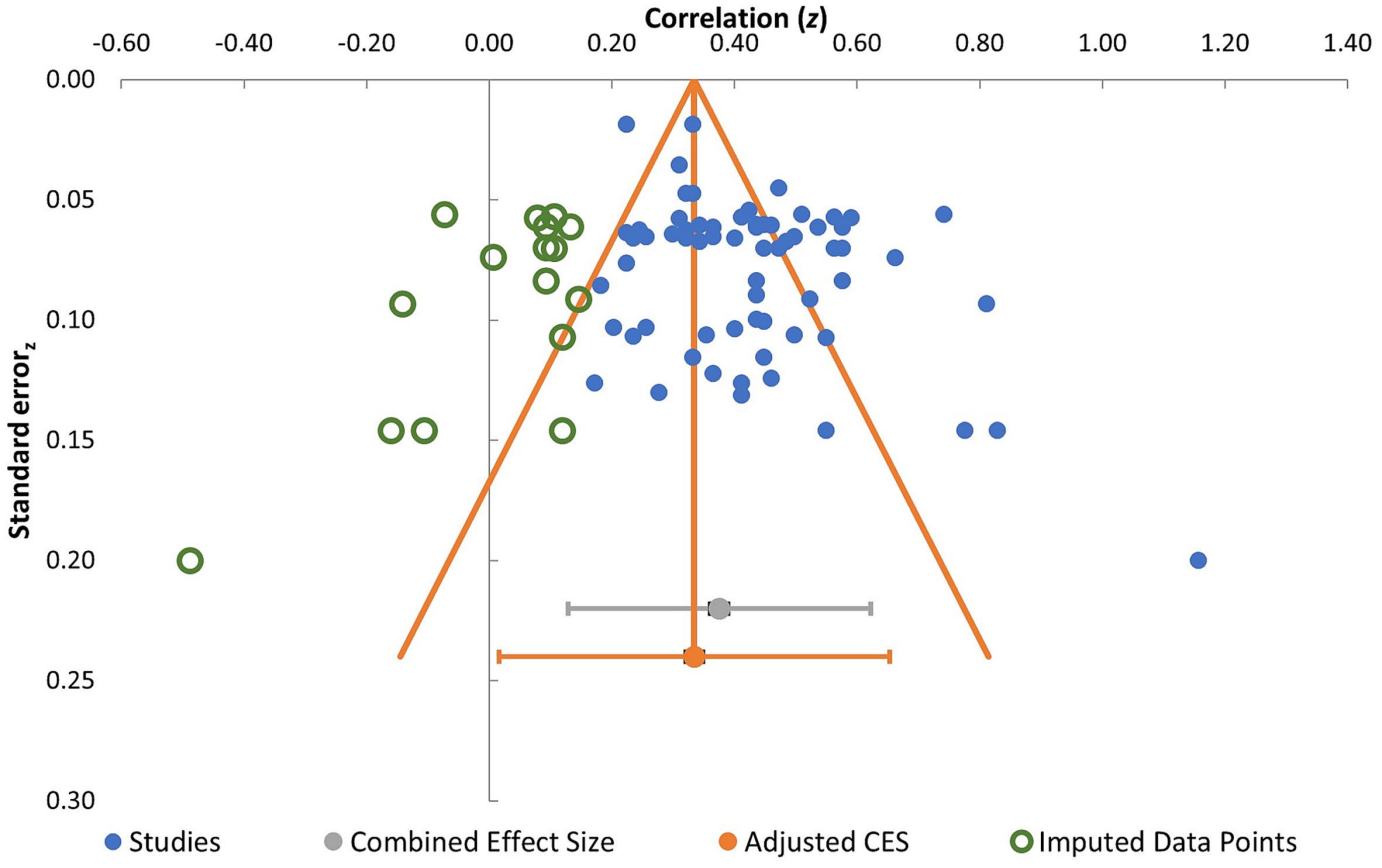
Funnel plot for inspection of symmetry of effect sizes around the combined effect size.

The funnel plot is presented in Figure 5, with correlation coefficients expressed as Fisher’s z. Visual inspection suggested asymmetry in the distribution of studies around the pooled effect size when the imputed studies were not considered. The trim-and-fill procedure imputed 16 studies to restore symmetry. After adjustment, the pooled effect size was 0.38 (95% CI [0.34, 0.41]), which was lower than the original estimate but remained within the medium effect size range. Taken together, these findings suggest evidence of publication bias, although the adjusted estimate remained broadly consistent with the original conclusion regarding the magnitude of the association.

## 4 Discussion

The present meta-analysis aimed to investigate the relationship between problem-solving appraisal, as assessed by the PSI, and internalizing disorders, specifically depression and anxiety. This analysis incorporated data from 49 studies, and there were several salient findings. First, the effect size of the relationship between problem-solving appraisal and internalizing disorders was found to be medium. This finding signifies that individuals who appraise their problem-solving abilities negatively tend to exhibit higher levels of internalizing disorders and supports cognitive model predictions that self-appraisals are a central factor in the onset and maintenance of mental health disorders.

The present study findings regarding self-appraisal are comparable to study findings regarding self-efficacy, as both constructs refer to an individual’s belief in their ability to manage and execute the actions necessary to achieve specific outcomes. Both problem-solving appraisal and self-efficacy involve self-perceptions of competence, which are critical in coping with stressors. Lonigro and colleagues (Lonigro et al., 2023) reported that emotional self-efficacy (i.e., appraisal of one’s emotional competence in regulating affective states) impacted internalizing behavior among adolescents, while Lau and colleagues (Lau et al., 2023) found self-appraisal biases among children with social anxiety. Karr and White (Karr and White, 2024) reported that lower academic self-efficacy was associated with higher levels of anxiety and depression among university students. Existing research has also highlighted the role of problem-solving appraisal in mental health outcomes (Chow and Chan, 2010; Padmanabhanunni and Pretorius, 2023).

The second major finding of this study is that age was not a significant moderator of the relationship between problem-solving appraisal and depression and anxiety, respectively. This finding suggests that the association between problem-solving appraisal and internalizing disorders is consistent across age groups. This finding is important because it implies that interventions aimed at improving problem-solving appraisal could be beneficial across a broad age range. Problem-solving therapy, which involves training in adaptive problem-solving attitudes and skills, has been effective in treating common mental health disorders (Zhang et al., 2018; Shang et al., 2021). Additional training in modifying dysfunctional appraisals of problem-solving competencies could be incorporated into such interventions. Interventions can specifically target these maladaptive appraisals to help individuals develop a more positive and realistic view of their problem-solving abilities. These positive self-appraisals can then lead to increased confidence and motivation in engaging in problem-solving activities, which may further reduce symptoms of depression and anxiety.

Third, the analysis found that the combined effect sizes were similar for studies that used the original English version of the PSI and those that used a translated version. Both groups of studies reported a medium effect size, which suggests a moderate relationship between problem-solving appraisal and internalizing disorders, regardless of the language of PSI administration. The lack of significant differences in effect sizes between studies that used translated contrasted with studies that used the original version of the PSI underscores the robustness of the PSI as a tool for assessing problem-solving appraisal across different languages and cultural contexts, potentially validating its cross-cultural applicability.

Fourth, the analysis found that the combined effect sizes were similar for studies that examined the relationship between problem-solving appraisal and anxiety and those that focused on the relationship between problem-solving appraisal and depression. There was no significant difference between these two combined effect sizes. This result indicates that problem-solving appraisal is equally associated with both anxiety and depression, reinforcing the notion that problem-solving appraisal is salient in the context of various internalizing disorders.

Fifth, after accounting for effect size dependencies the results indicated that there was no statistically significant difference between the adjusted and original combined effect sizes. This consistency in the combined effect size suggests that the overall findings of the meta-analysis are robust and the observed relationship between problem-solving appraisal and internalizing disorders is not influenced by dependencies among effect sizes within studies. This finding strengthens the reliability of the results and supports the conclusion that problem-solving appraisal has a moderate and consistent association with internalizing disorders.

Finally, the findings confirmed the presence of publication bias, such that studies with larger effect sizes were more likely to be published, potentially inflating the overall effect size reported in the meta-analysis. The issue of publication bias in meta-analytic research has been well documented, and its detection has important implications for interpreting the results of this meta-analysis (McShane et al., 2016). While the overall effect size remains in the medium range, the presence of bias suggests that the true effect size may be slightly smaller than initially reported. This finding highlights the need for cautious interpretation of meta-analytic findings and the importance of considering publication bias when evaluating the strength of the relationship between problem-solving appraisal and internalizing disorders.

An important contextual consideration is that a substantial proportion of the included studies were conducted with student samples. The observed association between problem-solving appraisal and internalizing disorders may therefore be influenced by the characteristics of younger and more educated populations, among whom the nature and consequences of negative problem-solving appraisals may differ from those observed in broader community or clinical groups.

This study has several limitations. The included studies exhibited significant heterogeneity in terms of sample characteristics, methodologies, and measures of internalizing disorders, and this variability may influence the generalizability of the findings. The majority of the studies included in the meta-analysis were cross-sectional, which limits the ability to draw causal inferences about the relationship between problem-solving appraisal and internalizing disorders. Longitudinal studies are needed to better understand the directionality and causality of this relationship. Most of the included studies relied on self-reported measures to assess problem-solving appraisal and internalizing disorders. Self-report instruments are subject to biases such as social desirability and response style, which can affect the accuracy of the data. A further limitation is that a large proportion of the included studies involved student populations. This may restrict the generalizability of the findings, as the relationship between problem-solving appraisal and internalizing disorders may differ across age groups, educational backgrounds, and clinical versus non-clinical populations.

## 5 Conclusion

In conclusion, this meta-analysis provides evidence for a moderate relationship between problem-solving appraisal and internalizing disorders. However, the presence of publication bias suggests that the observed effect size may be inflated. These findings underscore the importance of problem-solving appraisal in the context of mental health. It suggests that negative problem-solving appraisal may represent a useful intervention target in cognitive-behavioral and problem-solving-based approaches, particularly through strategies aimed at restructuring maladaptive self-appraisals and strengthening confidence in coping and problem resolution. The study also highlights the need for ongoing efforts to address publication bias in meta-analytic research.
